# Vascular endothelial growth factor mediates the therapeutic efficacy of mesenchymal stem cell-derived extracellular vesicles against neonatal hyperoxic lung injury

**DOI:** 10.1038/s12276-018-0055-8

**Published:** 2018-04-13

**Authors:** So Yoon Ahn, Won Soon Park, Young Eun Kim, Dong Kyung Sung, Se In Sung, Jee Yin Ahn, Yun Sil Chang

**Affiliations:** 1 Department of Pediatrics, Samsung Medical Center, Sungkyunkwan University School of Medicine, Seoul, South Korea; 2 Stem Cell and Regenerative Medicine Institute, Samsung Medical Center, Seoul, South Korea; 3 Department of Health Sciences and Technology, SAIHST, Sungkyunkwan University, Seoul, South Korea; 4 Department of Molecular Cell Biology, Samsung Medical Center, Sungkyunkwan University School of Medicine, Suwon, South Korea; 5 Single Cell Network Research Center (MRC), Sungkyunkwan University School of Medicine, Suwon, South Korea

## Abstract

We previously reported the role of vascular endothelial growth factor (VEGF) secreted by mesenchymal stem cells (MSCs) in protecting against neonatal hyperoxic lung injuries. Recently, the paracrine protective effect of MSCs was reported to be primarily mediated by extracellular vesicle (EV) secretion. However, the therapeutic efficacy of MSC-derived EVs and the role of the VEGF contained within EVs in neonatal hyperoxic lung injury have not been elucidated. The aim of the study was to determine whether MSC-derived EVs attenuate neonatal hyperoxic lung injury and, if so, whether this protection is mediated via the transfer of VEGF. We compared the therapeutic efficacy of MSCs, MSC-derived EVs with or without VEGF knockdown, and fibroblast-derived EVs in vitro with a rat lung epithelial cell line challenged with H_2_O_2_ and in vivo with newborn Sprague-Dawley rats exposed to hyperoxia (90%) for 14 days. MSCs (1 × 10^5^ cells) or EVs (20 µg) were administered intratracheally on postnatal day 5. The MSCs and MSC-derived EVs, but not the EVs derived from VEGF-knockdown MSCs or fibroblasts, attenuated the in vitro H_2_O_2_-induced L2 cell death and the in vivo hyperoxic lung injuries, such as impaired alveolarization and angiogenesis, increased cell death, and activated macrophages and proinflammatory cytokines. PKH67-stained EVs were internalized into vascular pericytes (22.7%), macrophages (21.3%), type 2 epithelial cells (19.5%), and fibroblasts (4.4%) but not into vascular endothelial cells. MSC-derived EVs are as effective as parental MSCs for attenuating neonatal hyperoxic lung injuries, and this protection was mediated primarily by the transfer of VEGF.

## Neonatal lung damage: Vesicles as vehicles for treatment

Membrane-bound sacs called vesicles, secreted by stem cells, carry a protein that might prevent oxygen damaging the lungs of newborn infants. This damage is particularly prevalent in babies born prematurely. The ‘mesenchymal’ stem cells generate cells of connective tissue, blood and blood vessels. They produce vascular endothelial growth factor (VEGF), a protein known to allow the stem cells to protect against neonatal lung injuries caused by oxygen. In studies with rats, Jee-Yin Ahn, Yun Sil Chang and colleagues at Sungkyunkwan University in South Korea, found secreted vesicles carrying VEGF are as effective as the whole stem cells. They also confirmed that the effect was specifically due to the VEGF. The research extends understanding of the protective action of VEGF and suggests the vesicles should be explored for their therapeutic potential in newborn infants.

## Introduction

Bronchopulmonary dysplasia (BPD) is a chronic lung disease that occurs in infancy and results from prolonged ventilator and oxygen treatment. Despite recent advances in neonatal intensive care medicine, BPD remains a major cause of mortality and morbidity in premature infants, with few clinically effective treatments^1,2^. Therefore, new effective therapies for BPD are urgently needed.

Previously, we and others have reported that mesenchymal stem cell (MSC) transplantation or MSC-conditioned medium significantly attenuates neonatal hyperoxic lung injuries in preclinical animal BPD models, and this protective effect was predominantly mediated by paracrine rather than regenerative mechanisms^3–10^. Moreover, the feasibility and short- and long-term safety of allogenic MSC transplantation in preterm neonates have been reported in a recent phase I clinical trial of MSC administration for BPD prevention with a 2-year follow-up in infants^11,12^. However, concerns remain regarding the tumorigenicity and other side effects of transplanting viable MSCs^13^.

Extracellular vesicles (EVs) are a nuclear membrane vesicles secreted by a variety of cells, 40–100 nm in diameter that contain numerous proteins, lipids, and RNAs, similar to those present in the originating cells; these EVs transport extracellular messages and mediate cell-to-cell communication^14–18^. Recently, MSC-derived EVs were shown to mediate the therapeutic efficacy of MSCs in various disorders, such as cardiovascular disease^19^, lung injury^13,20^, acute kidney injury^21^, fetal hypoxic ischemic brain injury^22^, and hypoxic pulmonary hypertension^20,22^, through the transfer of mRNA, miRNA, and proteins^20,21,23,24^. The use of MSC-derived EVs is a promising new therapeutic modality for BPD, since this therapy is cell-free and thus may bypass concerns associated with viable MSC treatment. Nevertheless, the therapeutic efficacy of MSC-derived EVs for BPD is unclear.

In this study, we evaluated whether the intratracheal transplantation of MSC-derived EVs is as effective as MSCs alone in a newborn rat model of hyperoxic lung injuries and, if so, whether this protection is mediated primarily through mRNA and protein transfer from the EVs to the injured lung tissue. We specifically examined the transfer of vascular endothelial growth factor (VEGF), as we previously identified a critical role for MSC-secreted VEGF in attenuating hyperoxic lung injuries in neonatal rats^9^.

## Materials and methods

### Mesenchymal stem cells

Human umbilical cord blood (UCB)-derived MSCs from a single donor at passage 6 were obtained from Medipost Co., Ltd. (Seoul, Korea). Human fibroblasts (MRC5; No. 10171) were purchased from the Korean Cell Line Bank (Seoul, Korea).

### Isolation of EVs

EVs were collected from the cell culture supernatant. After seeding 5 × 10^6^ MSCs per plate and culturing the cells to confluency in 100-mm plates, the cells were washed and then serum-starved for 6 h in conditioned media (α-MEM, Gibco, Grand Island, NY, USA). The conditioned media were centrifuged at 3000 r.p.m. for 30 min at 4 °C (Eppendorf, Hamburg, Germany) to remove cellular debris, followed by centrifugation at 100,000 r.p.m. for 120 min at 4 °C (Beckman, Brea, CA, USA) to sediment the EVs. The total EV protein content was quantified by measuring the protein concentration using the Bradford assay. Details are described in online supplement.

### VEGF-knockdown EVs

To knockdown VEGF, MSCs were transfected with siRNA targeting VEGF using Lipofectamine (Invitrogen, Carlsbad, CA, USA), as described in online supplement^9^. As a negative control, scramble siRNA was transfected into MSCs using the same method. Next, EVs were obtained from the conditioned media of the VEGF siRNA- or scramble siRNA-transfected MSCs.

### Animal model

Normoxic rat pups were kept in room air, and hyperoxic rat pups were raised with their dams in hyperoxic chambers (90% oxygen) from birth until postnatal day (P) 14. Newborn rats were randomly divided into 7 experimental groups: the normoxia control group (a, *n* = 18), hyperoxia control group (b, *n* = 18), hyperoxia with MSCs group (c, *n* = 19), hyperoxia with non-transfected MSC-derived EVs group (d, *n* = 18), hyperoxia with scramble siRNA-transfected MSC-derived EVs group (e, *n* = 18), hyperoxia with VEGF siRNA-transfected MSC-derived EVs group (f, *n* = 19), and hyperoxia with fibroblast-derived EVs group (g, *n* = 19). At P5, 5 × 10^5^ naive MSCs in 50 µL of saline, 20 µg of EVs derived from naive MSCs, scramble siRNA-transfected MSCs, VEGF siRNA-transfected MSCs or fibroblasts in 50 µL of saline, or an equal volume of saline (for the control group) were administered intratracheally.

### Labeling and localization of donor EVs

Details regarding the antibodies and image evaluation are described in online supplements. After the intratracheal injection of EVs labeled with a PKH67 green fluorescent dye at P5, the immunofluorescence localization of donor EVs was assessed in lungs were collected at P6. Pulmonary cells, including type 2 alveolar cells; total macrophages; activated macrophages; vascular smooth muscle cells; vascular endothelial cells; and vascular pericytes were labeled with red fluorescent antibodies in the lungs at P6. The co-localization rate of the donor EVs in each host pulmonary cell type was evaluated.

### Statistical analyses

The data are expressed as the means ± standard error of the mean (SEM). For continuous variables, statistical comparisons among groups were performed by one-way analysis of variance and Tukey’s post hoc analysis. All data were analyzed using SPSS version 18.0 (SPSS, Inc., Chicago, IL, USA). *P* < 0.05 was considered statistically significant.

## Results

### Characterization of MSC-derived EVs

**Fig. 1:**
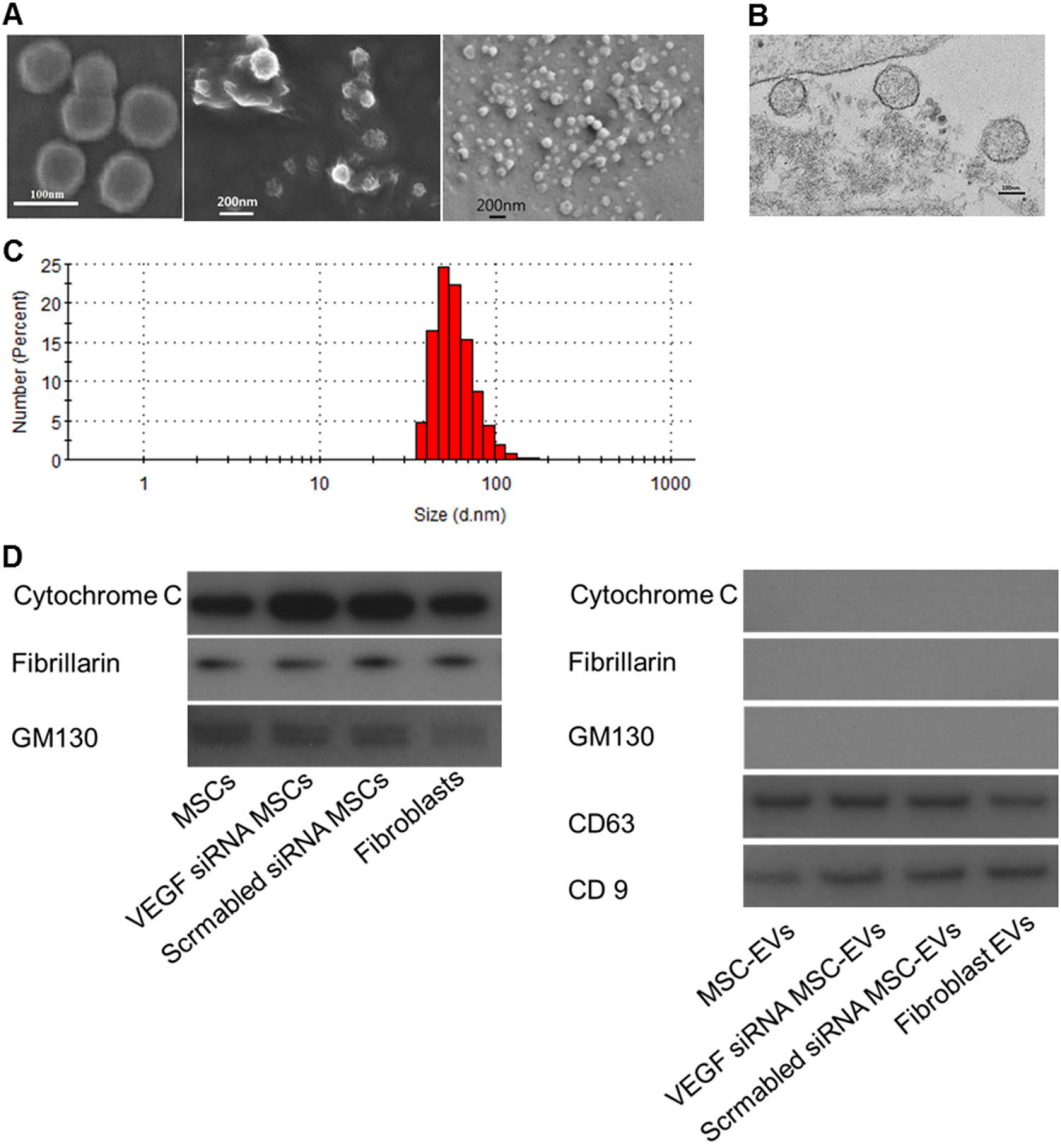
Confirmation of extracellular vesicles (EVs). **a** Scanning electron microscopy images of the EVs derived from mesenchymal stem cells (MSCs). **b** Transmission electron microscopy images of EVs derived from mesenchymal stem cells (MSCs). **c** Particle size distribution (number-weighted size distribution) of MSC-derived EVs. **d** Western blot assays of MSCs and MSC-derived EVs; cytochrome C (mitochondria marker), fibrillarin (nucleus marker), GM130 (Golgi apparatus marker), CD63, and CD9 (exosome marker)

Cell death was not indicated by terminal deoxyribonucleotidyl transferase-mediated dUTP-digoxigenin nick-end labeling (TUNEL) assays in cultured cells prior to EV isolation (data not shown). Scanning and transmission electron microscopy images (Fig. 1a, b, and Supplemental Fig. 1) indicated that isolated EVs were spheroidal in shape with a peak size distribution of 50–70 nm in diameter, as measured by nanoparticle tracking analysis (Fig. 1c), and the EVs were positive for EV-specific CD63 and CD9 markers, as determined by western blotting (Fig. 1d). Moreover, mitochondrial, nuclear, and Golgi apparatus markers were observed in cells but not in EVs (Fig. 1d).

### VEGF knockdown and human and rat VEGF levels

**Fig. 2:**
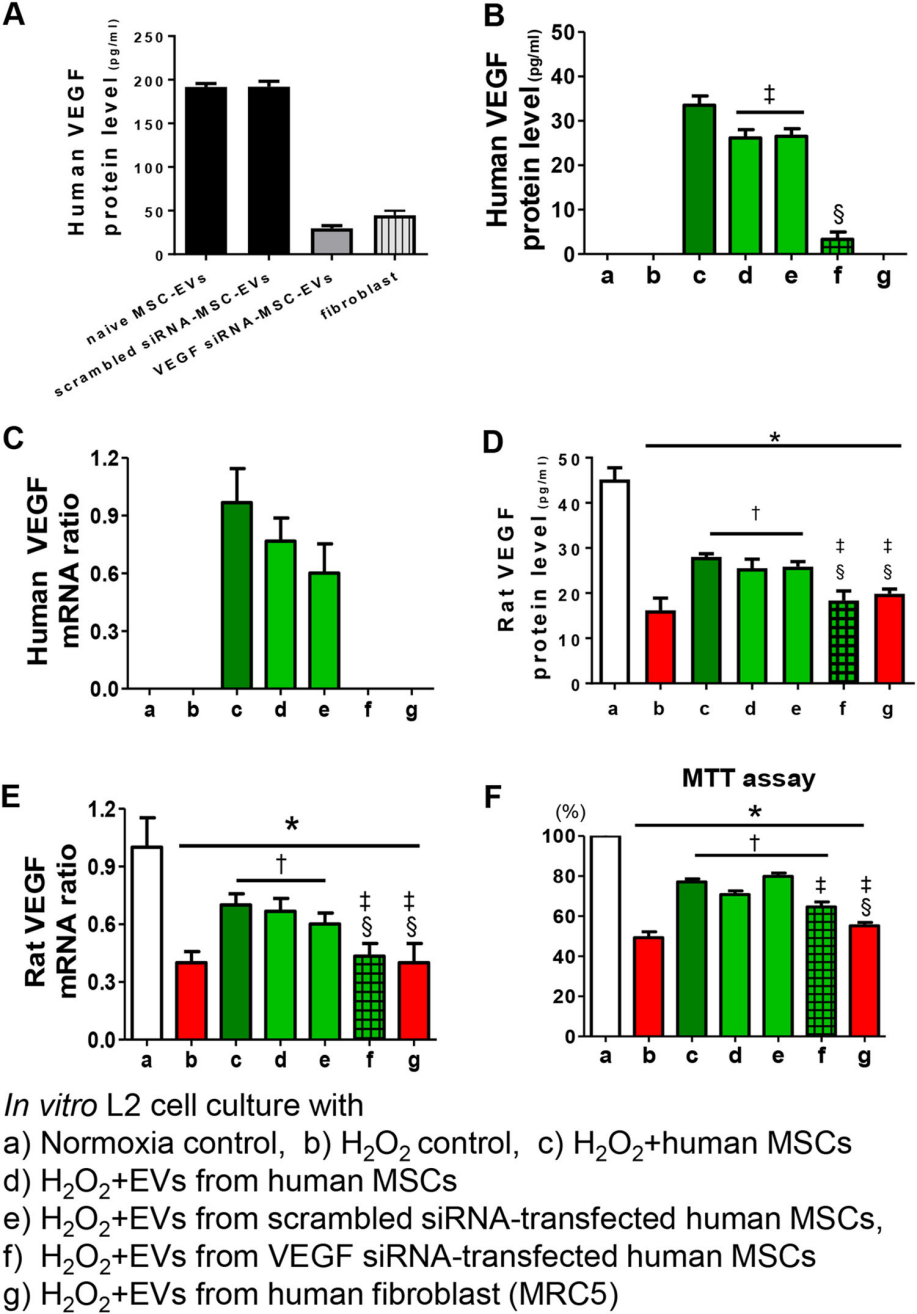
Induction of VEGF expression in rat lung epithelial L2 cells by the VEGF from MSC-derived extracellular vesicles (EVs) rescues oxidative injury in vitro. **a** The VEGF levels were measured in the EVs derived from naive mesenchymal stem cells (MSCs), the EVs from scramble siRNA-transfected MSCs, the EVs from VEGF siRNA-transfected MSCs, and the EVs from fibroblasts. The EVs from VEGF siRNA-transfected MSCs or fibroblasts presented with substantially decreased VEGF levels compared to the levels in EVs from naive MSCs or scramble siRNA-transfected MSCs. Rat lung epithelial (L2) cells were treated with H_2_O_2_ for 1 h to induce oxidative stress. L2 cells were co-treated with naive MSCs, EVs from naive MSCs, EVs from scramble siRNA-transfected MSCs, EVs from VEGF siRNA-transfected MSCs, or EVs from fibroblasts. In the culture medium, the expression of human VEGF protein (**b**), human VEGF mRNA (**c**), rat VEGF protein (**d**), and rat VEGF mRNA (**e**) was measured. **f** The cell survival rate in each group was evaluated by 3-(4,5-dimethylthiazol-2-yl)-2,5-diphenyltetrazolium bromide (MTT) assays. The in vitro L2 cell culture groups are as follows: a, normoxic control; b, H_2_O_2_ control; c, H_2_O_2_ + human MSCs; d, H_2_O_2_ + EVs from human MSCs; e, H_2_O_2_ + EVs from scramble siRNA-transfected human MSCs; f, H_2_O_2_ + EVs from VEGF siRNA-transfected human MSCs; and g, H_2_O_2_ + EVs from human fibroblast (MRC5). Data are presented as the means ± SEM. *P < 0.05 compared to the normoxic control, ^†^*P* < 0.05 compared to the H_2_O_2_ control, ^‡^*P* < 0.05 compared to H_2_O_2_ + human MSCs, ^§^*P* < 0.05 compared to H_2_O_2_ + EVs from human MSCs

VEGF protein levels were measured by enzyme-linked immunosorbent assay (ELISA) in EVs from VEGF siRNA-transfected MSCs or fibroblasts and were significantly reduced compared to those in the non-transfected naive or scramble siRNA-transfected MSC-derived EVs (Fig. 2a).

Human- and rat-specific VEGF protein and mRNA expression levels were measured by ELISA and PCR, respectively, in cultured rat L2 cells after H_2_O_2_ exposure with or without human MSCs or MSC-derived EVs.

No human-specific VEGF protein or mRNA was detected in the supernatant of the normoxic or H_2_O_2_-exposed rat L2 cells cultured alone or co-cultured with fibroblast-derived EVs (Fig. 2b, c). The human VEGF protein levels in the H_2_O_2_-exposed rat L2 cell supernatant were highest when co-cultured with MSCs, high when co-cultured with non-transfected or scramble siRNA-transfected MSC-derived EVs, and lowest when co-cultured with VEGF siRNA-transfected MSC-derived EVs, respectively. Human VEGF mRNA was detectable only following co-culture with MSCs, with non-transfected MSC-derived EVs or with scramble siRNA-transfected MSC-derived EVs but not with VEGF siRNA-transfected MSC-derived EVs.

The significant decrease in the rat VEGF protein and mRNA levels in the H_2_O_2_-exposed control group compared to the normoxic control group was significantly improved by co-culture with MSCs, with non-transfected MSC-derived EVs, or with scramble siRNA-transfected MSC-derived EVs but not with VEGF siRNA-transfected MSC-derived or fibroblast-derived EVs (Fig. 2d, e).

### Protection of MSC-derived EVs in vitro

In the cytotoxicity assay of cultured rat L2 cells, the H_2_O_2_ exposure significantly reduced cell survival compared to that under normoxic conditions (Fig. 2f). H_2_O_2_-induced cell death was significantly improved by co-culture with MSCs, with non-transfected MSC-derived EVs or with scramble siRNA-transfected MSC-derived EVs but not with VEGF siRNA-transfected MSC-derived or fibroblast-derived EVs.

To determine whether MSC-derived EVs show a dose-dependent pattern of protective effects, the in vitro model described above was treated with different amounts of MSC- derived EVs (1, 5, 10, 15, and 20 µg). The oxidative stress-induced increase in cell death was reduced in the MSC- derived EV-treated groups at all doses, except in the 1-µg MSC-derived EV group (Supplemental Fig. 2). Compared to the 5-µg MSC-derived EV group, the 10-, 15-, and 20-µg MSC-derived EV groups presented with significantly improved protective effects, and there were no differences among the groups (Supplemental Fig. 2).

In general, MSCs are believed to release both soluble VEGF and EV-associated VEGF. Here we tried to assess the amount of VEGF released in EV-associated form and attempted to compare the protective potency against oxidative stress-induced cell death between the soluble and EV-associated VEGF forms. First, we assessed the ratio of EV-associated VEGF to the total amount of VEGF released from MSCs in the culture media. The EV-associated VEGF form comprised 46.8% (Supplemental Fig. 3a) of the total VEGF present, and we therefore suggest that approximately half of the VEGF is released in the EV-associated form and the remaining half of the VEGF is released in the soluble form from MSCs. In addition, the VEGF amount was measured in the MSC-derived EVs. In the 10-µg MSC-derived EV group, the measured VEGF level was 1.9 ng/ml. In the cytotoxicity assay of cultured rat L2 cells exposed to H_2_O_2_, the cells were treated with 10 µg of MSC-derived EVs and 1.9 ng of VEGF. The level of oxidative stress-induced cell death was significantly improved with the MSC-derived EV and VEGF treatments (Supplemental Fig. 3b). Moreover, compared with the recombinant VEGF-treated group, the MSC-derived EV-treated group presented with greater protective effects (Supplemental Fig. 3b).

### Protection of MSC-derived EVs in vivo

**Fig. 3:**
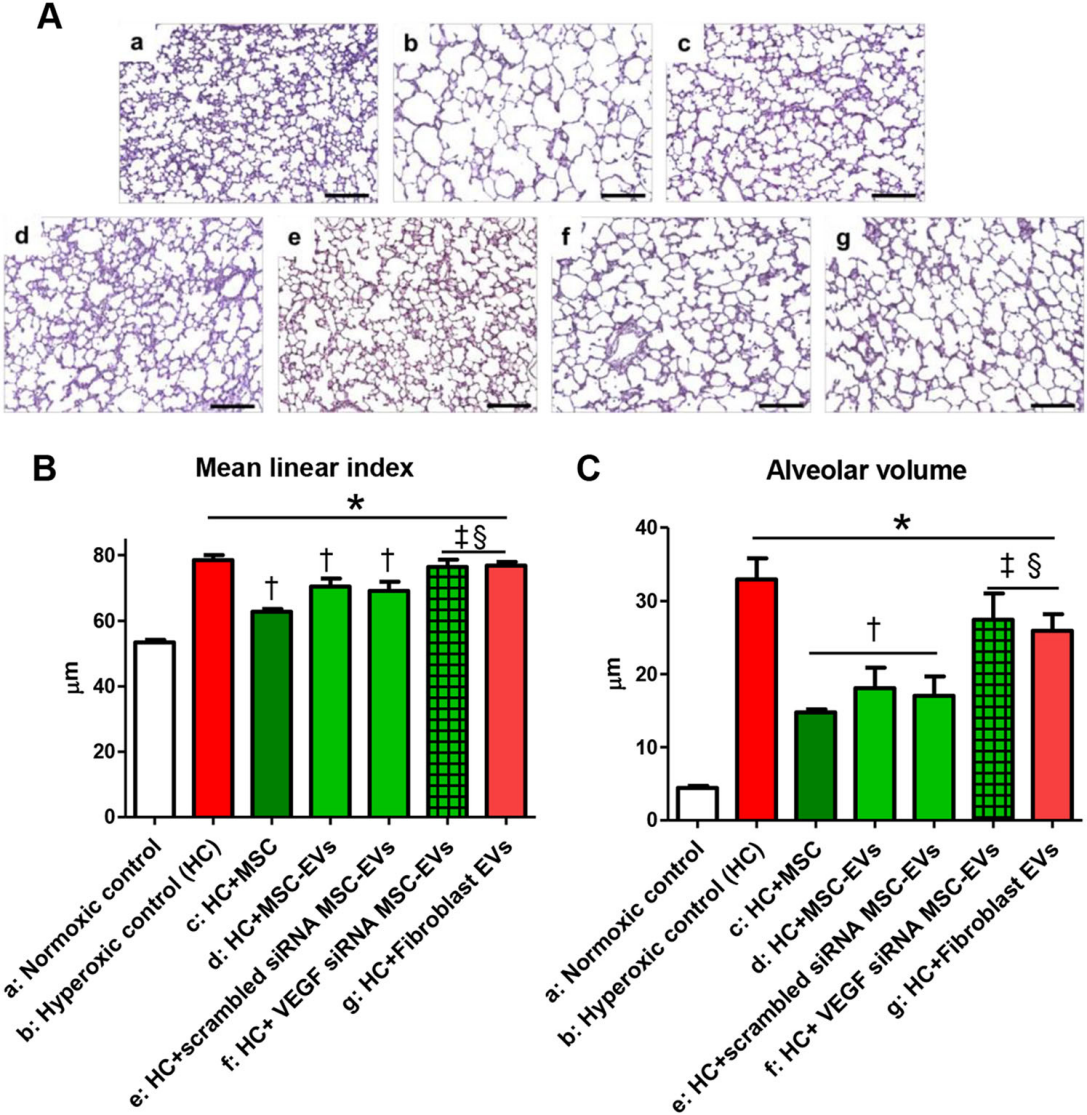
Extracellular vesicles (EVs) released from MSCs attenuated hyperoxia-induced impaired alveolarization in the lung tissue partly by transferring VEGF. **a** Representative photomicrographs of hematoxylin and eosin staining in the lungs of P14 rats from each group (scale bar = 25 μm). The degree of alveolarization was assessed by the (**b**) mean linear intercept and (**c**) mean alveolar volume. The EV injection dose per rat was 20 µg. The experimental in vivo groups are as follows: a, normoxic control; b, hyperoxic control; c, hyperoxia + MSCs; d, hyperoxia + MSC-derived EVs; e, hyperoxia + scramble siRNA-transfected human MSC-derived EVs; f, in vivo hyperoxia + VEGF siRNA-transfected human MSCs EVs; and g, in vivo hyperoxia human fibroblast (MRC5)-derived EVs. Data are presented as the means ± SEM. *P < 0.05 compared to the normoxic control, ^†^*P* < 0.05 compared to the hyperoxic control, ^‡^*P* < 0.05 compared to hyperoxia + human MSCs, ^§^*P* < 0.05 compared to hyperoxia + EVs derived from human MSCs

**Fig. 4:**
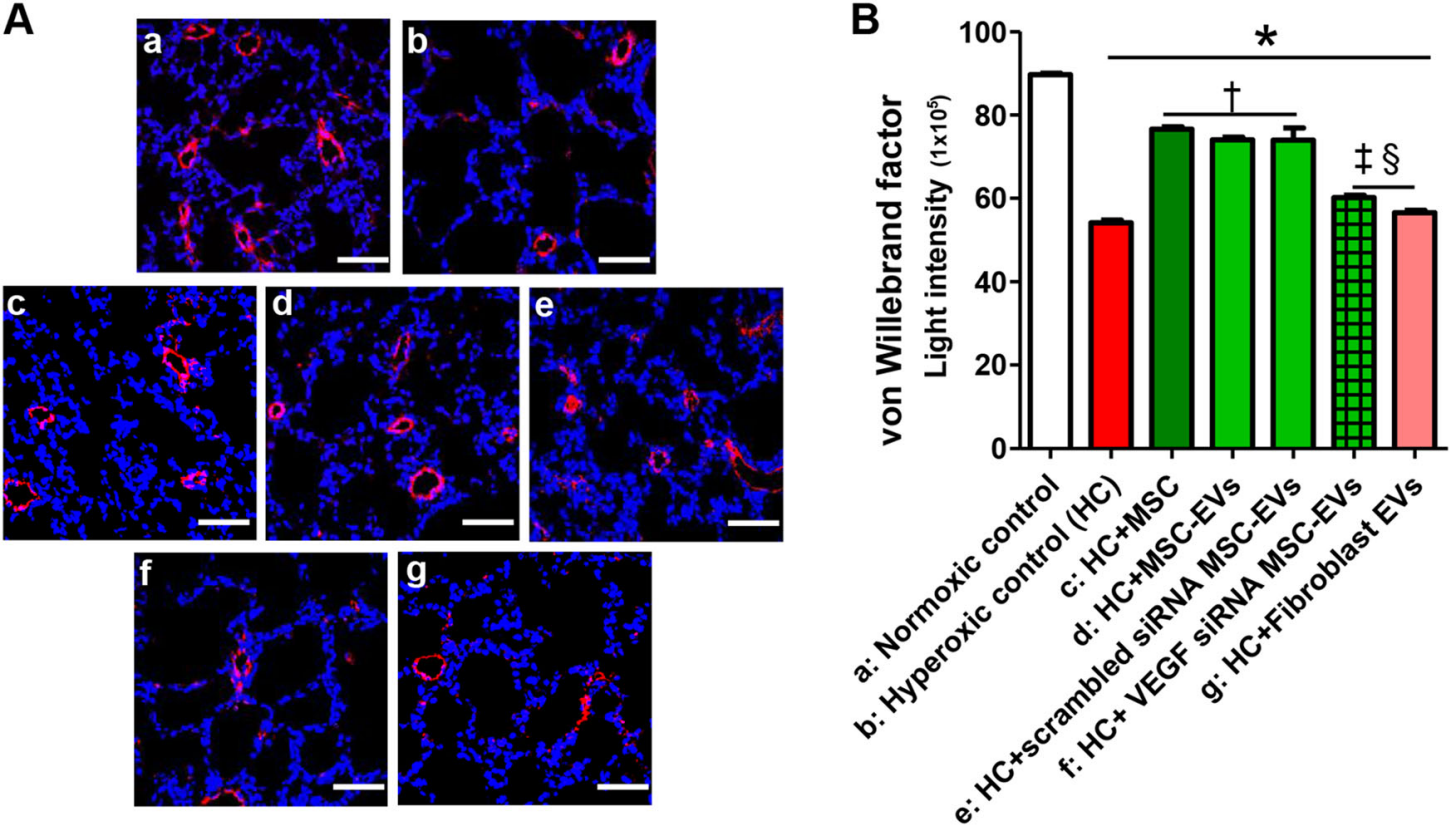
Extracellular vesicles (EVs) released from MSCs improved the hyperoxia-induced impaired pulmonary vascularization partly via transferring VEGF. Pulmonary angiogenesis was determined by staining for vWF in histological sections of P14 rat lungs. **a** Representative immunofluorescence photomicrographs of vWF staining in the lungs of P14 rats in each group. Nuclei were labeled with 4′,6-diamidino-2-phenylindole (DAPI, blue), and vWF was labeled with the fluorescent marker 5(6)-carboxyfluorescein diacetate *N*-succinimidyl ester (CFSE, red) (×200, scale bar = 50 μm). **b** The mean light intensity of vWF immunofluorescence per high power field (HPF) in each group. The EV injection dose was 20 µg per rat. The experimental in vivo groups are as follows: a, normoxic control; b, hyperoxic control; c, hyperoxia + MSCs; d, hyperoxia + MSC-derived EVs; e, hyperoxia + scramble siRNA-transfected human MSC-derived EVs; f, in vivo hyperoxia + VEGF siRNA-transfected human MSCs-derived EVs; and g, in vivo hyperoxia human fibroblast (MRC5)-derived EVs. Data are presented as the means ± SEM. *P < 0.05 compared to the normoxic control, ^†^*P* < 0.05 compared to the hyperoxic control, ^‡^*P* < 0.05 compared to hyperoxia + human MSCs, ^§^*P* < 0.05 compared to hyperoxia + EVs derived from human MSCs

In the rat lung tissue, the mean linear index and alveolar volume were significantly increased (Fig. 3), indicating impaired alveolarization, and the expression of von Willebrand factor (vWF) was significantly reduced (Fig. 4), indicating impaired angiogenesis, in the hyperoxic group compared to the normoxic group. The hyperoxia-induced impaired alveolarization and angiogenesis were significantly improved by the intratracheal transplantation of MSCs and non-transfected or scramble siRNA-transfected MSC-derived EVs but not by VEGF siRNA-transfected MSC-derived or fibroblast-derived EV transplantation.

**Fig. 5:**
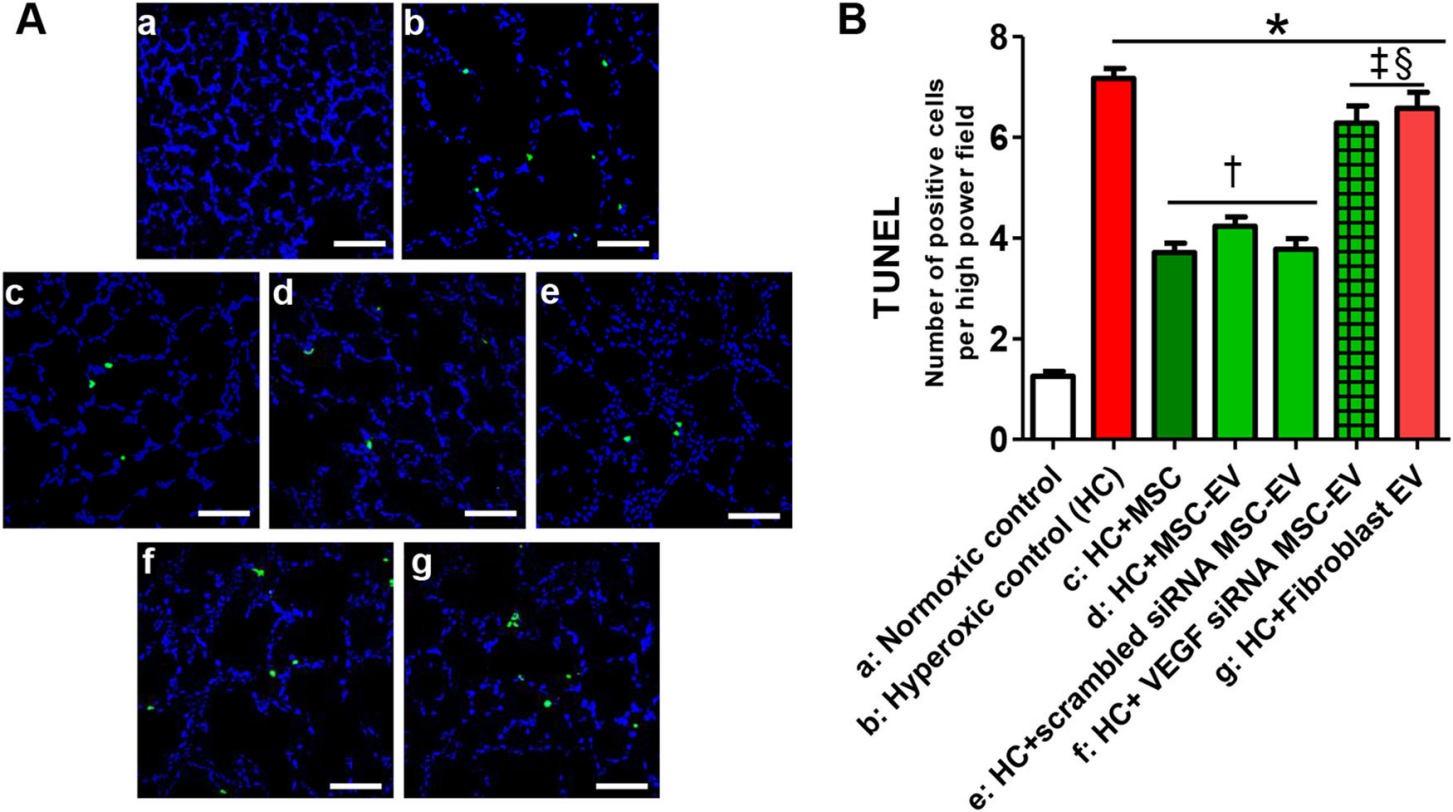
Extracellular vesicles (EVs) released from MSCs improved hyperoxia-induced cell death partly via VEGF. **a** Representative optical microscopy photomicrographs of TUNEL-stained lung histological sections from P14 rats in each group. The nuclei are labeled with DAPI (blue), and the TUNEL-positive cells are labeled with FITC (green) (×200, scale bar = 50 μm). **b** The average number of TUNEL-positive cells per high-power field (HPF) in each group. The EV injection dose was 20 µg per rat. The experimental in vivo groups are as follows: a, normoxic control; b, hyperoxic control; c, hyperoxia + MSCs; d, hyperoxia + MSC-derived EVs; e, hyperoxia + scramble siRNA-transfected human MSC-derived EVs; f, in vivo hyperoxia + VEGF siRNA-transfected human MSCs-derived EVs; and g, in vivo hyperoxia human fibroblast (MRC5)-derived EVs. Data are presented as the means ± SEM. *P < 0.05 compared to the normoxic control, ^†^*P* < 0.05 compared to the hyperoxic control, ^‡^*P* < 0.05 compared to hyperoxia + human MSCs, ^§^*P* < 0.05 compared to hyperoxia + EVs derived from human MSCs

The hyperoxia-induced increase in the number of TUNEL-positive cells in the rat lung tissue was significantly attenuated by the intratracheal transplantation of MSCs and non-transfected or scramble siRNA-transfected MSC-derived EVs but not by VEGF siRNA-transfected MSC-derived or fibroblast-derived EV transplantation (Fig. 5).

### Effects of MSC-derived EVs on the inflammatory response in vivo

**Fig. 6:**
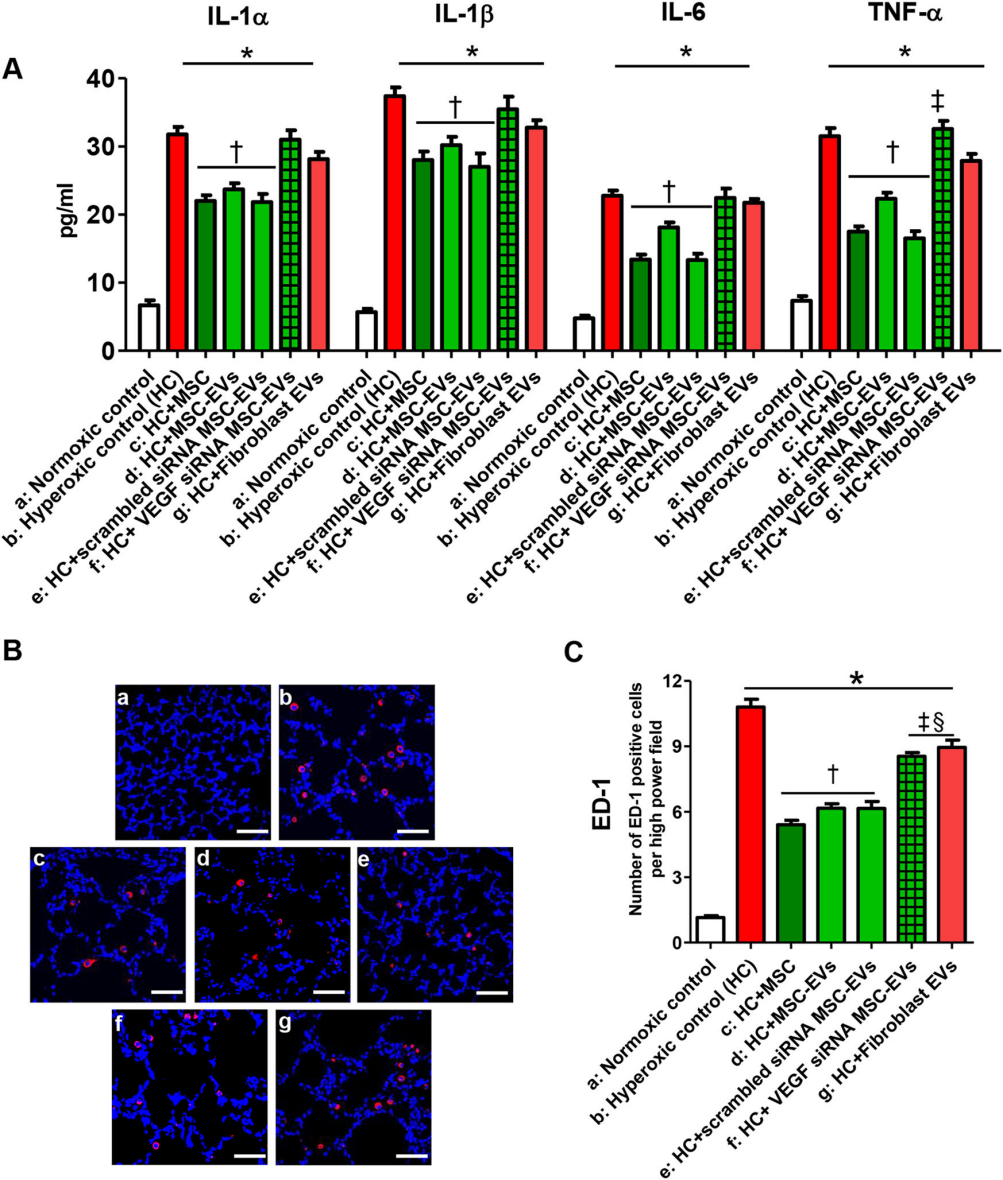
Extracellular vesicles (EVs) released from MSCs attenuated the hyperoxia-induced increase in the inflammatory response in lung tissue partly via transferring VEGF. **a** The expression of interleukin (IL)-1α, IL-1β, IL-6, and TNF-α in P14 rat lungs from each group was measured with ELISA. **b** Representative immunofluorescence photomicrographs of ED-1 staining, which indicates active macrophages in the lungs of the P14 rats from each group. ED-1 positive alveolar macrophages were labeled with CFSE (red), and nuclei were labeled with DAPI (blue) (×200, scale bar = 50 μm). **c** The average number of ED-1 positive cells per HPF in each group. The EV injection dosage was 20 µg per rat. The experimental in vivo groups are as follows: a, normoxic control; b, hyperoxic control; c, hyperoxia + MSCs; d, hyperoxia + MSC-derived EVs; e, hyperoxia + scramble siRNA-transfected human MSC-derived EVs; f, in vivo hyperoxia + VEGF siRNA-transfected human MSCs-derived EVs; and g, in vivo hyperoxia human fibroblast (MRC5)-derived EVs. Data are presented as the means ± SEM. *P < 0.05 compared to the normoxic control, ^†^*P* < 0.05 compared to the hyperoxic control, ^‡^*P* < 0.05 compared to hyperoxia + human MSCs, ^§^*P* < 0.05 compared to hyperoxia + EVs derived from human MSCs

In the rat lung tissue, the levels of inflammatory cytokines, such as interleukin (IL)-1α, IL-1ß, IL-6, and tumor necrosis factor-α (Fig. 6a), as well as ED-1-positive alveolar macrophages (Fig. 6b, c) were significantly increased in the hyperoxic group compared to the normoxic group. These hyperoxia-induced increases in the inflammatory cytokine and ED-1-positive alveolar macrophage levels were significantly attenuated by the intratracheal transplantation of MSCs, non-transfected MSCs, or scramble siRNA-transfected MSC-derived EVs but not by VEGF siRNA-transfected MSC-derived or fibroblast-derived EVs.

### Localization of donor MSC-derived EVs in vivo

**Fig. 7:**
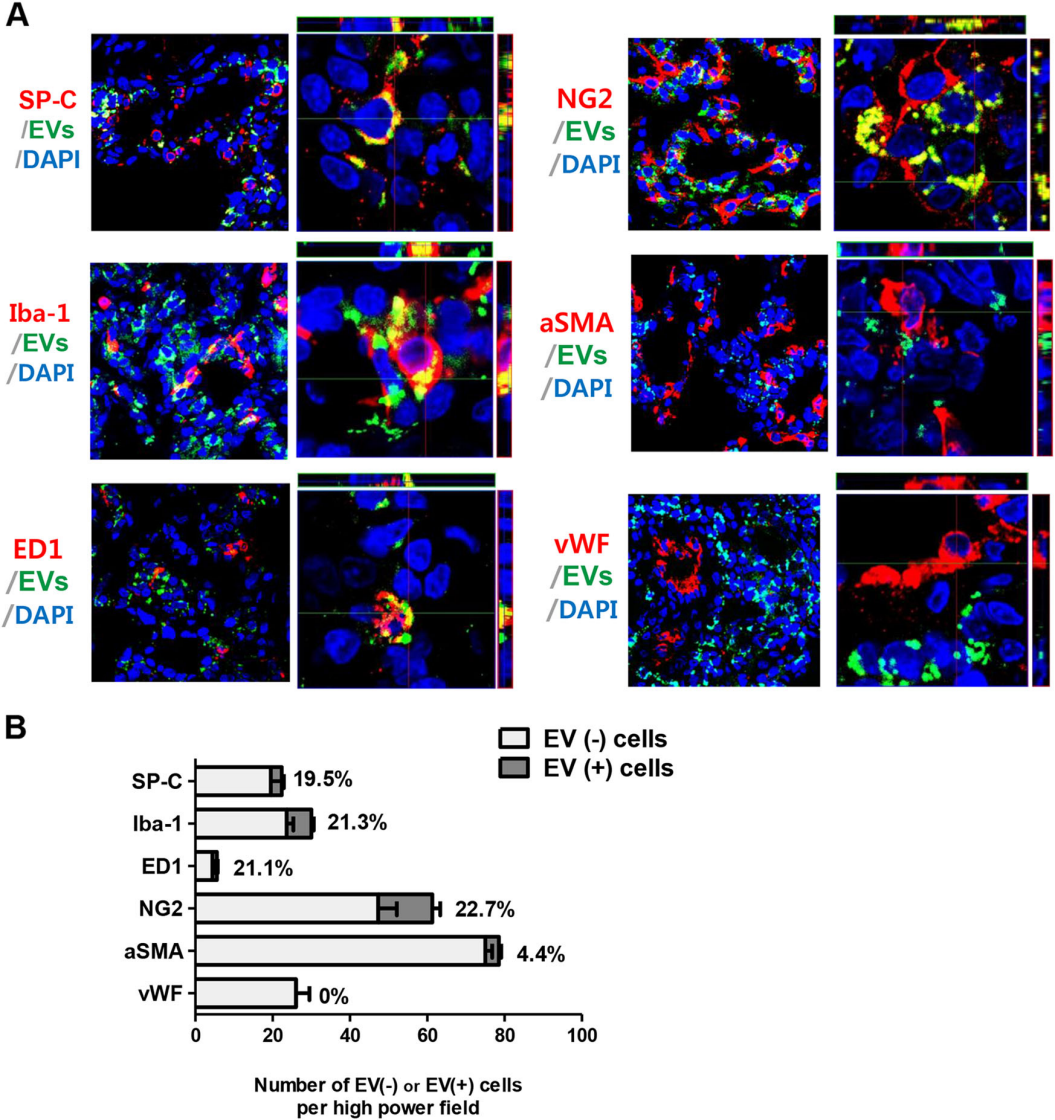
Distribution of the intratracheally delivered extracellular vesicles (EVs) according to the pulmonary tissue cell type. MSC-derived EVs displayed a cell-type dependent distribution in the pulmonary tissue. **a** Representative immunofluorescence photomicrographs of donor EVs and host pulmonary cell staining in the lungs of P14 rats from each group. The nuclei are labeled with DAPI (blue), and the type 2 alveolar cells, total macrophages, activated macrophages, vascular smooth muscle cells, vascular endothelial cells, and vascular pericytes were each immunohistochemically stained with pro-surfactant protein C (SP-C), Iba-1, ED1, α-smooth muscle actin, von Willebrand factor (vWF), and NG2 (red), respectively. The extracellular vesicles (EVs) were prestained with PKH67 dye (green). The three-dimensional images of the co-localized donor EVs and recipient cells were obtained using confocal *z*-stack images. **b** The number of each type of host pulmonary cell was counted and evaluated according to EVs co-localization. The rate of donor EVs incorporation into each type of host pulmonary cell is presented as the percentage of double-labeled pulmonary cells with EVs among the non-EVs-merged pulmonary cells. EVs: Extracellular vesicles

To determine the distribution of transplanted MSC-derived EVs in the lung tissue in vivo, the co-localization of PKH67 green fluorescence with various lung cell markers was examined by immunofluorescence staining at P6 (Fig. 7). PKH67 green fluorescence most frequently co-localized with NG-2-positive pericytes (22.7%) and was also identified with Iba-1-positive (21.3%) and ED-1 (21.1%)-positive alveolar macrophages, SP-C-positive type II pneumocytes (19.5%), and α-smooth muscle actin-positive smooth muscle cells (4.4%) but not with vWF-positive vascular endothelial cells.

## Discussion

In this study, we isolated MSC-secreted EVs that were <100 nm in diameter and that were positive for exosome-specific CD9 and CD63 markers from conditioned media. MSC-derived but not fibroblast-derived EVs were as effective as parental MSCs in attenuating both H_2_O_2_-induced cell death in rat lung epithelial L2 cells in vitro and hyperoxic lung injuries in vivo, suggesting that the protective effects of EVs are specific to the parental MSCs from which EVs are derived. Although the protective effects of both MSCs and their conditioned media against neonatal hyperoxic lung injuries have been previously reported^25^, this is the first study demonstrating that MSC-derived EVs are as effective as MSCs in attenuating neonatal hyperoxic lung injuries. These findings suggest that MSC-derived EVs are key paracrine therapeutic mediators of MSCs and thus indicates their potential for use in safe and effective cell-free therapy^26^ for the neonatal lung disorder BPD without theoretical tumorigenic potential.

Previously, we observed a significantly lower level of human VEGF protein in VEGF siRNA-transfected MSCs but not in scramble siRNA-transfected MSCs for up to 7 days after transfection^9^. In this study, the human VEGF protein level was significantly lower in the VEGF siRNA-transfected MSC-derived MSCs than in the non-transfected or scramble siRNA-transfected MSC-derived EVs. Overall, these findings indicate that VEGF knockdown in MSCs by siRNA transfection was effective and specific not only in the parental MSCs but also in the MSC-derived EVs.

Recently, we reported that the VEGF secreted by transplanted MSCs may be a critical paracrine factor that plays a seminal role in attenuating neonatal hyperoxic lung injuries, such as impaired alveolarization and angiogenesis, increased cell death, and inflammatory responses^9^. In the present study, the protective effects of MSC-derived EVs observed in L2 cells exposed to H_2_O_2_ in vitro and against in vivo hyperoxic lung injuries in newborn rats were abolished in VEGF siRNA-transfected MSC-derived EVs but not in scramble siRNA-transfected MSC-derived EVs. Taken together, these results suggest that the VEGF mRNA and protein within MSC-derived EVs are critical paracrine factors responsible for protecting against neonatal hyperoxic lung injuries.

The precise mechanisms by which human VEGF protein and mRNA within MSC-derived EVs preserve rat VEGF protein and mRNA and protect neonatal lung tissue against hyperoxic injuries remain unclear. Despite the absence of human VEGF ELISA cross-reactivity kit with rat VEGF protein, a <85% homology^27^ and similar functional mechanisms of action^28,29^ have been observed between human and rat VEGF. Since VEGF is the essential survival factor in the lung alveolar and vascular endothelium^30,31^, our results revealing that H_2_O_2_-induced cell death in rat lung epithelial L2 cells in vitro in the hyperoxia control group may be primarily attributable to the significant reduction in the rat VEGF protein and mRNA levels below the threshold level for survival. Moreover, in this study, detectable human VEGF protein and mRNA levels were observed in the non-transfected MSC-derived EV co-culture group, and the rat VEGF protein and mRNA levels were significantly increased in this group compared to the hyperoxia control group, which correlated well with the simultaneous significant attenuation of H_2_O_2_-induced cell death in the L2 cells. Following co-culture with VEGF siRNA-transfected MSC-derived EVs but not with scramble siRNA-transfected MSC-derived EVs, the human VEGF protein levels were significantly reduced, human VEGF mRNA was undetectable, and the rat VEGF protein and mRNA levels were significantly reduced compared to those in the non-transfected MSC-derived EV co-culture group; in addition, the protective effects of the MSC-derived EVs against H_2_O_2_-induced cell death were simultaneously abrogated. Overall, these findings suggest that the human VEGF mRNA and protein in the MSC-derived EVs contribute to the restoration of rat VEGF protein and mRNA levels, ultimately leading to improved survival directly by enhancing EV production and indirectly by maintaining VEGF levels within the local microenvironment above the threshold level essential for rat lung tissue survival against hyperoxic injury^32^.

Although, the VEGF level was significantly increased in the group co-cultured with MSC-derived EVs compared to the hyperoxia control group, the VEGF level was still much lower in this co-culture group than in the normoxia group. Although VEGF overexpression in MSCs has been reported to significantly enhance stem cell-mediated therapeutic efficacy in neural and cardiac repair^33,34^, no studies have investigated whether the transplantation of VEGF-overexpressing MSC-derived EVs enhance the beneficial effects of MSCs in this BPD model. Therefore, further studies are necessary to clarify these points.

The cytoprotective effects of EVs can be mediated by delivering cargo containing various proteins, mRNA, and miRNAs to target cells^21,24,35^. Previously, we observed the critical role of VEGF secreted by transplanted MSCs in mediating the protective effects of MSCs against hyperoxic lung injuries, such as the attenuation of impaired alveolarization and angiogenesis, the reduction of TUNEL-positive apoptotic cells and ED-1-positive cells, and the downregulation of pro-inflammatory cytokine levels^9^. In this study, the attenuated impairment of alveolarization and angiogenesis and the anti-apoptotic and anti-inflammatory effects that had been observed with the MSC-derived EV transplantation were abolished by the VEGF-knockdown MSC-derived EV transplantation. Overall, these findings suggest that the VEGF contained within the MSC-derived EVs is a critical paracrine factor that plays a seminal role in attenuating hyperoxic lung injuries in newborn rats.

The engulfment of EVs by target cells is necessary for the delivery of EV cargos and thus for communication between cells^36^. Therefore, our data showing the co-localization of EVs with alveolar macrophages, types II pneumocytes, and smooth muscle cells suggest that MSC-derived EV engulfment into host cells and subsequent communication are primarily responsible for mediating the anti-inflammatory and anti-apoptotic cytoprotective effects of EVs, as well as for improving alveolarization during neonatal hyperoxic lung injuries. However, our data showing that EVs do not co-localize with vascular endothelial cells despite the improved angiogenic effects are difficult to explain. Intratracheal EV delivery and the covering of vascular endothelial cells with pericytes may prevent EV engulfment into vascular endothelial cells. Recently, Franco et al.^37^ reported that pericytes are not only essential for angiogenesis but also protect vascular endothelial cells against cytotoxic stimuli, and pericyte-dependent survival signaling results from enforced paracrine and autocrine loops involving VEGF-A expression. Moreover, it has been suggested that differentiated pericytes express VEGF, which potentiates endothelial cell survival and the stability of microvessels^38,39^. In this study, EVs most frequently co-localized with pericytes. However, despite this engulfment into pericytes, the improved angiogenic effects of MSC-derived EVs were abrogated by VEGF knockdown. Overall, these findings suggest that the improved angiogenic effects of MSC-derived EVs are mediated not directly by engulfment into vascular endothelial cells but are indirectly induced by EV engulfment into pericytes and via pro-survival cross-talk between pericytes and endothelial cells; these findings highlight the critical role of the VEGF protein and mRNA contained within EVs for improved angiogenesis. Further studies are necessary to clarify these points.

In this study, the intratracheal transplantation of 20 µg of MSC-derived EVs at P5 was arbitrarily determined by reviewing the literature^20,23,26,40^, and our preclinical data from the MSC transplantation in the BPD animal model indicated that therapeutic efficacy was better with local intratracheal transplantation than with systemic intravenous or intraperitoneal administration^3^ at an early time point during hyperoxic lung injury^6^. However, further studies are necessary to confirm the optimal route, timing, and dose of MSC-derived EVs for transplantation.

In conclusion, MSC-derived EVs were as efficient as the parental MSCs in protecting against hyperoxic injuries both in vitro in a rat alveolar epithelial cell line and in vivo in newborn rats, and EV-transferred VEGF may act as a key paracrine factor, mediating cytoprotective effects to injured lung tissue.

## Electronic supplementary material

Supplemental Materials

Supplemental Fig. 1

Supplementary Fig. 2

Supplementary Fig. 3
