# Supplementary material for: Vascular endothelial growth factor mediates the therapeutic efficacy of mesenchymal stem cell-derived extracellular vesicles against neonatal hyperoxic lung injury
Source: Exp Mol Med. 2018 Apr 13;50(4):26. doi: 10.1038/s12276-018-0055-8 (PMC5938045; doi:10.1038/s12276-018-0055-8)
Supplement: Supplementary file 1 — Supplemental Materials [file 12276_2018_55_MOESM1_ESM.doc]

**Online Supplements**

**Vascular endothelial growth factor in mediating therapeutic efficacy of mesenchymal stem cell-derived extracellular vesicles against neonatal hyperoxic lung injury**

**MATERIALS AND METHODS**

***Cell preparation and culture conditions***

Differentiation potential and immunophenotypic study were performed as described in previous studies (1-6). Cells were cultured in Dulbecco's modified Eagle's medium (DMEM) with 10% fetal bovine serum (FBS) (Invitrogen, Carlsbad, CA, USA) at 37°C in a humidified atmosphere of 5% CO_2_. The culture medium was changed every 3–5 days.

***In vitro cell culture***

L2 cells (rat lung epithelial cells) were purchased from the Korea Cell Line Bank (Seoul, South Korea). L2 cells (rat lung epithelial cells, 5×10^3^ cells/well) were seeded into 96-well plates in 100 μl of RPMI 1640 medium containing 10% fetal bovine serum (FBS, Gibco, Grand Island, NY, USA) and cultured for 24 hours at 37 °C. To induce hyperoxic injury, cultured L2 rat lung epithelial cells were treated with 100 μM H_2_O_2_ for 60 min. Then, to confirm whether exotracellular vesicles (EVs) plays a role in the protective effects against cell death via transfer of VEGF, L2 rat lung epithelial cells were treated with H_2_O_2_ and supplemented with 1x10^3^ naïve mesenchymal stem cells (MSCs), 2ug/ml of EVs derived from naïve MSCs, from scrambled siRNA transfected MSCs, from VEGF siRNA transfected MSCs, or from fibroblasts in the top compartment of a Boyden chamber with identical manner above.

A colorimetric MTT [3-(4,5-dimethylthiazol-2-yl)-2,5-diphenyl tetrazolium] assay (Dojindo Molecular Technologies Inc, Gaithersburg, MD, USA) was used to assess cell viability according to the manufacturer’s protocol. Relative viabilities were determined by normalizing to 0% (no cells) and 100% (untreated cells) controls.

***In vitro VEGF silencing with siRNA***

To optimize siRNA silencing, we designed a 19-nt sequence-specific siRNA targeting VEGF (7). Sequences of the double-stranded siRNA targeting VEGF were as follows: sense: 5′-GGA GUA CCC UGA UGA GAU CdTdT-3′, antisense: 5′-GAU CUC AUC AGG GUA CUC CdTdT-3′. All chemically synthesized oligonucleotides including VEGF siRNA and scrambled siRNA were obtained from Samchully Pharmaceutical Company (Seoul, Korea). MSCs were transfected with siRNA oligonucleotides using Oligofectamine (Invitrogen, Carlsbad, CA, USA) according to the manufacturer's instructions. All assays or transplants were performed 24 h after RNA transfection as previously reported^1^. For negative control, scrambled siRNA was transfected into the MSCs using identical method.

***Isolation of extracellular vesicles***

After seeding of 5x10^6^ MSCs per plates, and culturing until confluent in 100-mm plates, cells were washed with phosphate buffered saline (PBS), and then cells were serum starved for 6 h in conditioned media (α-MEM, Gibco, Grand Island, NY, USA). EVs were collected from this culture supernatant of cells. Average protein amount of EVs obtained from the cell culture media per plate (seeded with 5x10^6^ MSCs) was about 20ug. Compared with MSCs, fibroblasts showed ten times less yield in releasing EVs. EVs were stored at −80°C, and frozen EVs were thawed at 37℃ and re-suspended with normal saline (4ug/ul) for further use.

***Confirmation of*** EVs

EVs were estimated by measuring Nanoparticle Tracking Analysis using Zetasizer ( Malvern Instruments Ltd, Malvern, UK). The obtained EVs was resuspended in PBS (500 μL, 1 mg/mL total protein), and characterized for size and polydispersity. Cells were fixed with 4% paraformaldehyde and 1% glutaraldehyde in 0.1 M sodium cacodylate buffer (pH 7.2) (Electron Microscopy Sciences, Hatfield, PA, USA) for 3 hours at room temperature, washed with PBS buffer, postfixed in 1% osmium tetroxide, progressively dehydrated in a graded ethanol series (50–100%), and embedded in Epon. Ultrathin (70- to 80-nm) sections were cut from the polymer with a Reichert (Depew, NY, USA) Ultracut S microtome and placed on copper grids. The grids briefly stained with uranyl acetate and observed under an electron microscope (H-7650, Hitachi, Tokyo, Japan). EVs were fixed with 2% paraformaldehyde, loaded on 300-mesh formvar/carboncoated electron microscopy grids (Electron Microscopy Sciences, PA, USA), post-fixed in 1% glutaraldehyde, and then contrasted and embedded. Transmission electron microscopy images were obtained with an FE-SEM microscope (SUPRA 55VP, Zeiss, Oberkochen, Germany).

***Localization of donor*** EVs

EVs were labeled with a PKH67 Green Fluorescent Cell Membrane Labeling Kit (Sigma-Aldrich, St. Louis, MO, USA) according to the manufacturer’s protocol. Immunofluorescence localization of donor EVs was performed on the 10µm thick cryostat sections at P6. The following primary antibodies were used as markers for type2 alveolar cells, total macrophages, activated macrophages, vascular smooth muscle cells, vascular endothelial cells and vascular pericytes: pro surfactant protein C (SP-C, 1:2000; Millipore), Iba-1 (1:200; Abcam, Cambridge, UK), ED1 (1:250; Millipore), Vimentin (1:150; Millipore), α-smooth muscle actin(α-SMA (1:200; Abcam), Von Willebrand factor (vWF,1:250; DAKO Carpinteria, CA, USA) and NG2 (1:200, Abcam), respectively. For each test, Alexa Fluor 568-labeled IgG were used as secondary antibodies (Invitrogen, Eugene, OR, USA). After red immunofluorescence labeling for each cell type markers, 3-dimentional images of co-localized donor EVs and recipient cells were obtained using confocal z-stack images. Single channel and merged confocal micrographs were captured using an LSM700 laser scanning confocal microscope (Carl Zeiss, Oberkochen, Germany) and the images were generated using ZEN 2009 Light Edition software (Carl Zeiss). The EVs and cells were considered to be double-labeled if co-labeling occurred with the relevant morphology in the x-z plane, which was produced by an orthogonal reconstruction from the z-series stacks taken with ×400 or ×800 magnification. In each pulmonary cell type, proportion of exosome incorportation was assessed by counting the cells using randomly selected seven high power fields (×200): the ratio of red and green co-merged cells among total red fluorescence positive counted cells.

***Animal model***

Animal procedures were approved by the Animal Care and Use Committee of Samsung Biomedical Research Institute, Seoul, Korea. This study was carried out in accordance with institutional and National Institutes of Health guidelines for laboratory animal care. Timed pregnant Sprague-Dawley rats (Orient Co., Seoul, Korea) spontaneously delivered rat pups. To avoid oxygen toxicity, the dams that were nursing the pups were rotated daily between litters in normoxic and hyperoxic conditions.

Cells, EVs, or saline was injected intratracheally with a 29-gauge needle. At P14, rat pups were sacrificed under deep pentobarbital anesthesia (60 mg/kg, intraperitoneal) and whole lung tissue was obtained for morphometric and biochemical analyses.

***Tissue preparation and morphometry***

Lungs were harvested after transcardiac perfusion with ice-cold PBS and fixed in 10% buffered formalin at room temperature or immediately snap-frozen in liquid nitrogen and stored at -80°C. For morphometric analysis, lungs were inflated with 10% buffered formalin instilled intratracheally in situ at a constant pressure of 20 cm H_2_O and fixed in the same fixative overnight at room temperature. After tissue processing, lungs were embedded in paraffin wax. Sections of 4-μm thickness were cut from the paraffin blocks and deposited on glass slides for hematoxylin-eosin staining. The slides were visualized using a Nikon Eclipse 80i microscope (Nikon Instruments Inc., Tokyo, Japan). The degree of alveolarization was estimated using the mean linear index (MLI) as previously described^2-4^. A minimum of three sections per sample and a minimum of six fields per section were evaluated randomly in a blinded manner.

***TUNEL assay***

Immunofluorescent terminal deoxynucleotidyl transferase dUTP nick end labeling (TUNEL) was performed to assess the degree of apoptosis in the lung as previously reported^1,5,6^. The number of TUNEL-positive cells was counted in 10 random, non-overlapping fields per slide at ×200 magnification by blinded examiners.

***Immunohistochemistry***

Paraffinized lung sections (5-μm thick) were deparaffinized, and angiogenesis was analyzed by immunofluorescent staining for von Willebrand factor (vWF) and alveolar macrophages (ED-1). Specimens were placed in a solution containing 0.1% (v/v) Triton X-100 and 0.5% (v/v) BSA in PBS and incubated with the following primary antibodies: anti-vWF (1:200; Dako, Glostrup, Denmark) and anti-monocyte/macrophage antibodies (1:100; anti-CD68 ED-1, mouse monoclonal, hemiproteincon, Millipore, MA, USA). Sections were stained with polyclonal rabbit anti-mouse immunoglobulins/fluorescein isothiocyanate (FITC) (1:200, Dako Uk Ltd) and polyclonal swine anti-rabbit immunoglobulins/FITC (1:200, Dako UK Ltd) for 2 h at room temperature. Vector shield mounting medium with DAPI (Vector Laboratories Inc.) was used to counterstain nuclei. A minimum of three sections per rat and six fields per each section were randomly assessed. To estimate vWF staining, the optical density of immunofluorescence was measured using Image J (National Institutes of Health, USA). The number of ED-1–positive cells was counted manually.

***Enzyme-linked immunosorbent assay (ELISA) and Reverse transcriptase polymerase chain reaction (RT-PCR)***

Frozen lungs were homogenized in cold buffer (50 mM Tris-HCl, pH 7.4) with 1 mM EDTA, 1 mM EGTA, 1 mM PMSF, 42 mM KCl, and 5 mM MgCl_2_ and centrifuged at 8,000 × g for 20 min at 4°C to remove cellular debris. The total protein content in the supernatant was determined using the Bradford method with a bovine serum albumin (Sigma-Aldrich) standard. Interleukin (IL)-1α, IL-1β, IL-6, and tumor necrosis factor (TNF)-α were measured using the Milliplex MAP ELISA Kit (Millipore) according to the manufacturer’s protocol.

In the culture media of rat L2 cells co-treated with MSC, EVs derived from naïve MSCs, scrambled siRNA transfected MSCs, or VEGF siRNA transfected MSCs, the amount of VEGF protein was determined using human- and rat-specific VEGF Quantikine ELISA kits (R&D Systems, Inc., Minneapolis, MN, USA) according to the manufacturer’s protocol.

RNA was isolated from L2 cell using the TRIZOL kit (Qiagen Inc., Valencia, CA, USA). The quality of the RNA was evaluated with the NanoDrop ND-1000 UV-Vis Spectrophotometer (NanoDrop Technologies, Wilmington,DE) according to the manufacturer’s instructions. The260/280 and 260/230 nm absorbance ratios of 1.8–2.0 implied a pure RNA sample. Custom-made primers for human VEGF, human GAPDH, rat VEGF and rat GAPDH were purchased (Bioneer Co., Daejeon, South Korea). The sequences were as follows: human VEGF(forward 5′- CCAATCGAGACCCTGGTG -3′; reverse5′- CACACAGGATGGCTTGAAGA -3′), RAT VEGF (forward 5′- ACAGAAGGGGAGCAGAAAGCCCAT -3′; reverse 5′- CGCTCTGACCAAGGCTCACAGT -3′), human glyceraldehyde 3-phosphate dehydrogenase (GAPDH) (forward 5 ′ -TGAGCGATGTGGCTCGGCT 3′ ; reverse 5′-CTCTCTGCTCCTCCTGTTCGAC-3′)and rat GAPDH (forward 5′- TCAACTACATGGTCTACATGTTCCAG -3′; reverse 5′- TCCCATTCTCAGCCTTGACTG -3′) . With SuperScript III One-Step RT-PCR System with Platinum Taq DNA Polymerase protocol from Invitrogen, RT-PCR was done according to manufacturer’s instructions. Amplified DNA products were run on a 1% agarose gel, and bands were visualized by an ethidium bromide. The expression level of each gene was semiquantified by densitometric analysis using Quantity One software (Bio-Rad, Hercules, CA, USA). The relative expression levels were estimated from the density ratio of the cytokines to glyceraldehyde 3-phosphate dehydrogenase (GAPDH, control).

**Supplemental figure legends**

Supplemental figure 1. Scanning electron microscopy images of extracellular vesicles (EVs) derived from vascular endothelial growth factor (VEGF) siRNA transfected mesenchymal stem cells (MSCs), scrambled siRNA-transfected MSC, and fibroblasts.

Supplemental figure 2. Dose-dependent protective efficacy of extracellular vesicles (EVs) released from MSCs. In the H_2_O_2_ treated L2 cells, cell survival rate was assessed by MTT assay after treatment of 1/5/10/15/20ug of MSCs-EVs. Data are presented as mean ± SEM. *P < 0.05 compared to normoxia +L2 cells, †P < 0.05 compared to H2O2+L2 cells, ‡P < 0.05 compared to H2O2+L2 cells+5ug of MSCs-EVs.

Supplemental figure 3. (A) The ratio of amount of VEGF measured in EV isolated from MSC culture media *versus* amount of VEGF measured in the whole culture media of MSCs was assessed using western blot analysis. (B) Protective efficacy of extracellular vesicles (EVs) released from MSCs and VEGF recombinant protein against oxidative stress induced cell death. From 10ug of EVs, measured VEGF level was 1.9ng. In the H_2_O_2_ treated L2 cells (lung epithelial cells), 10ug of EVs and 1.9ng of recombinant VEGF were treated. Data are presented as mean ± SEM. *P < 0.05 compared to normoxia +L2 cells, †P < 0.05 compared to H2O2+L2 cells, ‡P < 0.05 compared to H2O2+L2 cells+EVs.

**Supplemental references**

1. Chang YS, Ahn SY, Jeon HB, Sung DK, Kim ES, Sung SI *et al.* Critical role of vascular endothelial growth factor secreted by mesenchymal stem cells in hyperoxic lung injury. *Am J Respir Cell Mol Biol* 2014; **51:** 391-399.

2. Chang YS, Oh W, Choi SJ, Sung DK, Kim SY, Choi EY *et al.* Human umbilical cord blood-derived mesenchymal stem cells attenuate hyperoxia-induced lung injury in neonatal rats. *Cell Transplant* 2009; **18:** 869-886.

3. Chang YS, Choi SJ, Sung DK, Kim SY, Oh W, Yang YS *et al.* Intratracheal transplantation of human umbilical cord blood-derived mesenchymal stem cells dose-dependently attenuates hyperoxia-induced lung injury in neonatal rats. *Cell Transplant* 2011; **20:** 1843-1854.

4. Lee JH, Sung DK, Koo SH, Shin BK, Hong YS, Son CS *et al.* Erythropoietin attenuates hyperoxia-induced lung injury by down-modulating inflammation in neonatal rats. *J Korean Med Sci* 2007; **22:** 1042-1047.

5. Ahn SY, Chang YS, Sung DK, Yoo HS, Sung SI, Choi SJ *et al.* Cell type-dependent variation in paracrine potency determines therapeutic efficacy against neonatal hyperoxic lung injury. *Cytotherapy* 2015; **17:** 1025-1035.

6. Chang YS, Choi SJ, Ahn SY, Sung DK, Sung SI, Yoo HS *et al.* Timing of umbilical cord blood derived mesenchymal stem cells transplantation determines therapeutic efficacy in the neonatal hyperoxic lung injury. *PLoS One* 2013; **8:** e52419.
