# Supplementary figures and images for: Vascular endothelial growth factor mediates the therapeutic efficacy of mesenchymal stem cell-derived extracellular vesicles against neonatal hyperoxic lung injury
Source: Exp Mol Med. 2018 Apr 13;50(4):26. doi: 10.1038/s12276-018-0055-8 (PMC5938045; doi:10.1038/s12276-018-0055-8)

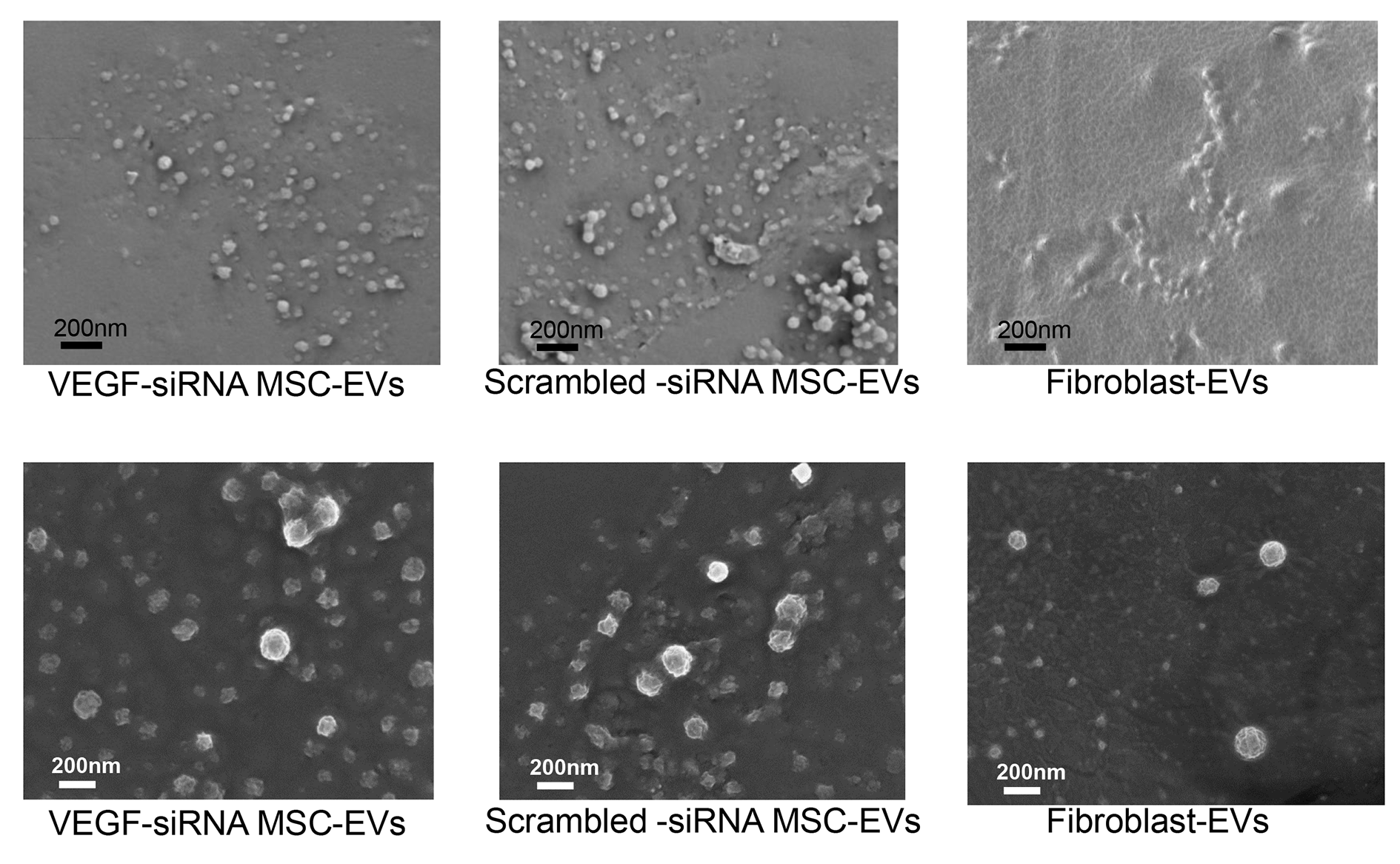

Supplement: Supplementary file 2 — Supplemental Fig. 1 [file 12276_2018_55_MOESM2_ESM.tif]

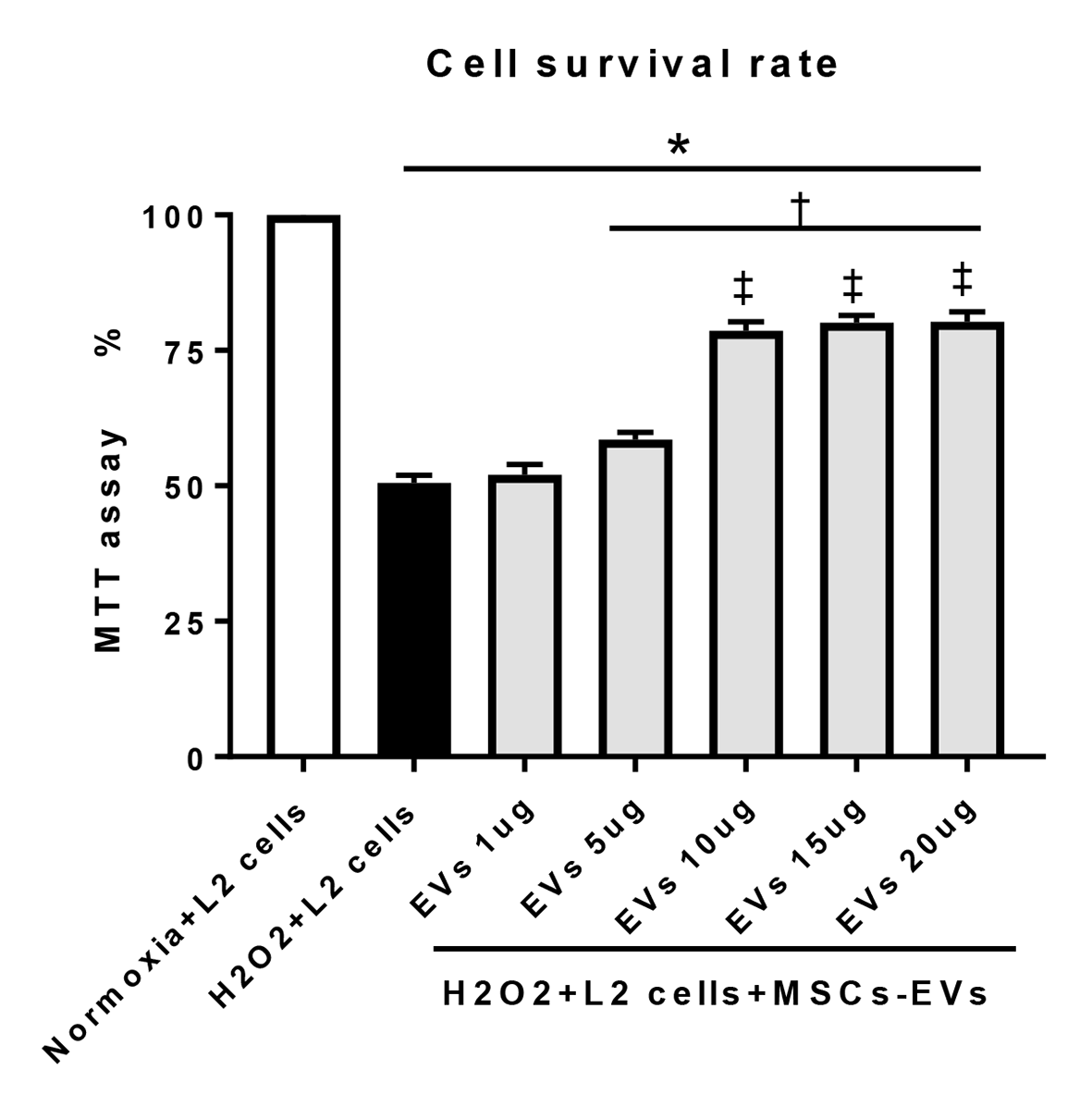

Supplement: Supplementary file 3 — Supplementary Fig. 2 [file 12276_2018_55_MOESM3_ESM.tif]

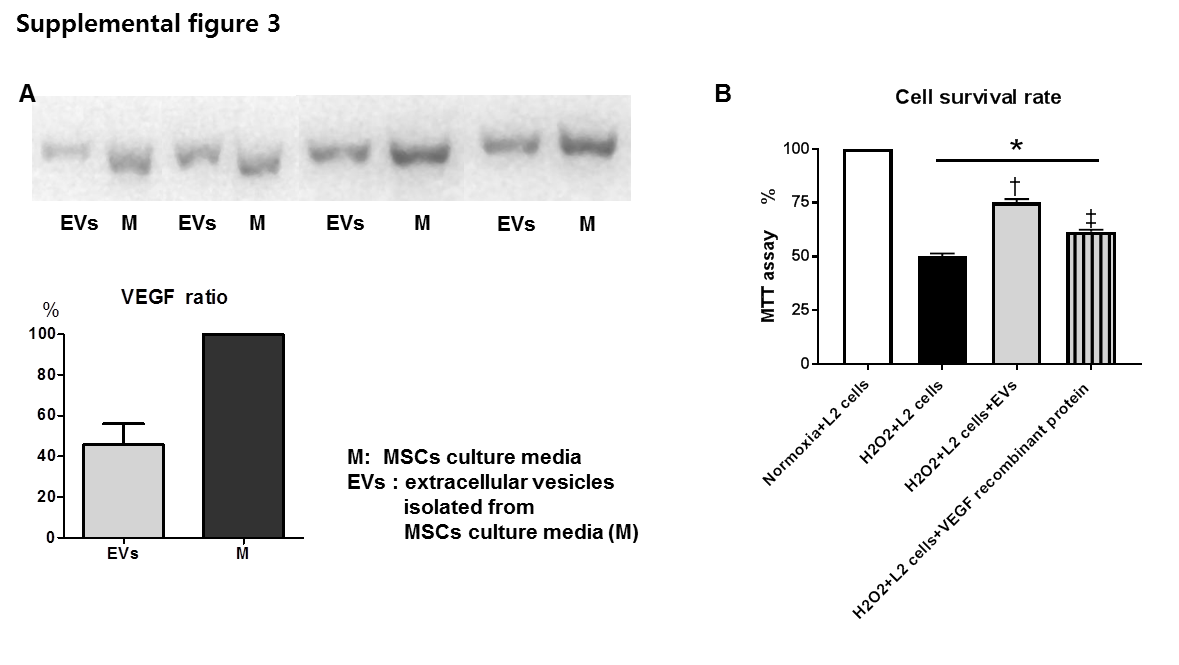

Supplement: Supplementary file 4 — Supplementary Fig. 3 [file 12276_2018_55_MOESM4_ESM.tif]
